# Psychological Predictors of COVID-19-Related Anxiety in Vulnerable Groups

**DOI:** 10.3390/ejihpe13090132

**Published:** 2023-09-14

**Authors:** Diana Bakalova, Ilina Nacheva, Tsvetelina Panchelieva

**Affiliations:** Department of Psychology, Institute for Population and Human Studies, Bulgarian Academy of Sciences, 1113 Sofia, Bulgaria; tsvetelina.p@iphs.eu

**Keywords:** COVID-19, vulnerable groups, fears, perceived stress, anxiety, resilience, optimism, pessimism

## Abstract

This study responds to the need to explore psychological predictors of COVID-19-related anxiety in vulnerable groups. An anonymous voluntary online survey was conducted (*n* = 520) with (a) working parents with young children (0–12 y.o.), (b) people with chronic physical conditions, (c) people with multiple vulnerability characteristics and (d) a control group (no self-reported vulnerability) in 2022. Findings showed that perceived stress of the parents and trait anxiety of the chronic sufferers were single weak positive predictors of COVID-19 anxiety. However, both psychological factors had a stronger effect on the pandemic-related anxiety for the group with multiple vulnerabilities. In the control group, trait resilience and optimistic expectations (combined with perceived stress) were moderate negative predictors of COVID-19 anxiety. The findings emphasize the importance of perceptions, expectations, trait anxiety as well as the need for intersectional research of vulnerability from multiple perspectives. Furthermore, they highlight the necessity of group-specific policies and interventions aimed both at handling the negative psychological tendencies of the vulnerable groups and at strengthening the positive tendencies of non-vulnerable groups, rather than tackling only emergent anxiety conditions in crisis times.

## 1. Introduction

The COVID-19 crisis deeply affected European societies and threw down a lot of challenges to the everyday practices of living. The pandemic brought about changes in beliefs and attitudes at the social, organizational and individual levels. These changes were related to special measures, concerning work regimes, public events, healthcare services and procedures, etc. The economic and social consequences, together with the health threat, became sources of fear, anxiety and stress for many people [1,2]. At the same time, the controversial and inconsistent explanations of reality—ranging from complicated scientific analyses to esoteric and conspiracy theories—aggravated this effect [1].

In this context, Bulgaria could be considered a specific or even an “exceptional” case [3] due to several reasons: it was the European country with the lowest level of gross domestic product (GDP) per capita in the EU in 2020, 2021 and 2022 [4]; it was the country that “exhibits the most extreme excess mortality figures” in Europe during the first two years of the Pandemic [5]. At the same time, the country reported the lowest COVID-19 immunization coverage [6] and the highest vaccine hesitancy [7] and refusal [8]. The crisis management started with a focus on prevention and restrictive measures at the very beginning [9]. The Bulgarian government formed a National Crisis Center responsible for organization and coordination of emergency planning and crisis management at the national level. Various preventive steps were set to limit infection including the closure of public areas, such as schools, universities, parks, concert and theater halls and sport venues, and temporary restrictions of movement [3]. Many private businesses were closed. Remote work regime was enforced, where possible, which was a completely unknown practice for employers in most of the sectors. The unprecedented measures were also related to the introduction of new educational modes—distant learning and virtual classrooms were fields, in which the school system lacked traditions and resources. The big changes in the daily routines required great flexibility and quick adaptation on both the community and individual levels. In this context, the crisis, caused by COVID-19, together with all related restrictions and consequences, became a source of anxiety because of the health (physical and mental), economic and social threats. As in all critical situations, there were groups in the society that were more vulnerable in regard to the wide range of risks and specific aspects of the situation [1].

The concept of vulnerability is complex, since it is “caused by structural social, economic and political determinants that disadvantage people” [10] (p. 153). It can also be understood as health inequities and social determinants of health [11]. From our perspective, it is very important to consider the mental health aspects and the impact of the crisis on them. Wirkner and colleagues bring evidence to the assumption that the pandemic can be regarded as being a “multidimensional stressor on mental health”, and in this context, some populations appear to be more vulnerable than others, even though the authors also point out that some inconsistencies arise [12] (p. 310). In our study, we approach vulnerability as a (potential) disadvantage in the COVID-19 crisis, which is related to various risk domains—psychological risks understood as role conflict and higher demands in the context of the COVID-19 crisis. Considering the specific Bulgarian context, including the introduction of the “stay at home” regime, remote work and innovative educational practices, we have identified **working parents with little children (0–12 y.o.)** as a vulnerable group. The selection is also in line with conclusions of a systematic review of the psychological literature on the topic [13] and with previous studies on the impact of COVID-19 in Bulgaria [14,15,16,17,18,19] and Italy [20]. Our focus is narrowed to families with babies and young children (in primary school), as they are particularly dependent on the schedule of healthcare and educational institutions. Growing older and gaining independence in adolescents are associated with relatively less parental stress, as evidenced by research [21,22].

As the pandemic was a health crisis at its core, the health-related risk domain was another perspective we considered in this study with the inclusion of **people diagnosed with chronic physical illness** as a separate vulnerable group. People with chronic conditions such as cardiovascular or respiratory diseases, diabetes, cancer, neurodegenerative conditions, overweight/obesity and/or any other physical conditions that compromise the immune system were found to be at higher risk of severe COVID-19 infection and mortality [23]. In addition, coping with a chronic condition often depends on the supply of certain medicines, as well as regular clinical examinations and procedures [24]. The selection is based on the higher levels of health risks for this particular group [25]. Participants diagnosed with mental disorders were excluded, since they have been regarded as a group, in which specific needs and experiences might appear under crisis conditions. In addition, the design of our survey required self-reporting and self-assessment that might not be applicable to patients diagnosed with certain mental disorders or experiencing acute psychotic symptoms [2].

When it comes to vulnerability, another important aspect is related to the effects of multiple factors that may affect individuals simultaneously. Therefore, we believe the concept must be approached from an intersectional and context-specific perspective. In order to gain a better understanding of nuances and to explore the role of psychological factors in times of crisis, we included in our study a **group with multiple vulnerability characteristics** such as individuals who are simultaneously parents of children 0–12 y.o. and chronic sufferers. To this group, we also added another parameter connected to economic risks in the pandemic, i.e., the loss of a job or business because of the measures related to COVID-19. Results from previous studies show that factors such as lower income and/or general insecurity are associated with higher levels of anxiety and depression [26]. In times of COVID-19, unemployment is related to more intense experiences of loss and fear [27]. Those who had lost their jobs due to the pandemic had the highest levels of stress and the worst control of their stress [28]. Interrupted work status is related to significantly higher levels of anxiety, depression and distress [29]. Pettinicchio and colleagues [30] found that worries about financial hardship, contracting COVID-19, along with the social isolation, were significantly associated with increased stress, anxiety and despair, especially in people with disabilities and chronic health conditions. In addition, data from Bulgaria show that economic risks were perceived as very important and even prevailed over the other risk domains from the very beginning of the crisis [31]. Therefore, we assume that parents of young children and chronic sufferers who lost their jobs, closed businesses or experienced significant changes in their financial status faced both economic and psychological challenges and can be considered vulnerable from multiple perspectives.

*Psychological predictors of COVID-19-related anxiety:* COVID-19 containment measures contributed to increased levels of emotional distress. Research conducted during the pandemic documented prevalence of mental health disorders, potentially arising from psychological stress associated with the pandemic outbreak. Among the possible predictors is anxiety, defined by the American Psychological Association (APA) [32] as “an emotion characterized by feelings of tension, worried thoughts and physical changes such as increased blood pressure”. Therefore, people who experience anxiety can have both psychological and physical problems. Stress is an individual’s adaptation response to internal or external threats [33], whereas perceived stress is the result of one’s appraisal of a stressor as threatening or nonthreatening, as well as their own abilities to cope (e.g., perceived efficacy or resources to respond to the threat). Perceived stress is defined as the extent to which situations and events in one’s life are appraised as unpredictable, uncontrollable and overwhelming [34]. In times of crisis and disaster, resilience acts as a buffer for crisis-related stress [35]. Resilience is defined as “the ability of a system to cope with change and especially sudden changes, while maintaining its functioning” [36] (p. 17). Resilience alleviates the development of anxiety and depression after adversity and crisis situations [37] and can be an important variable for interventions aimed at protecting the mental health of individuals against COVID-19-related stress, reducing COVID-19 stress/burnout [38,39]. On the other hand, perceptions and attitudes are potential sources of influence as well. Optimistic and pessimistic expectations act like powerful cognitive filters that change individual perceptions of the world and influence the way people adapt to unfamiliar situations and handle challenges and stressful events [40]. Optimism is seen as overall positive attitudes/expectations for the development of future events; a key ingredient of happiness [41,42]; and a cognitive, affective and motivational construct [43]. It is defined as a generalized tendency to expect favorable outcomes about the future rather than unfavorable ones [44]. Optimistic expectations (attitudes) determine the goals that individuals set for themselves, the efforts they invest, the response to certain events as well as the decisions they make [45]. Optimistic individuals tend to have positive expectations about the future, higher motivation, and hence, they are likely to cope better with stressful situations, which in turn, has a more favorable effect on their overall mental health, especially during COVID-19 [46]. The psychological literature suggests that optimism can serve as a buffer against psychological distress and anxiety and can foster resilience in adverse situations [47,48]. Research evidence in the pandemic times shows that there is a negative relationship between anxiety and optimism, since optimism could reduce anxiety and depression symptoms during critical situations [46,48,49]. Alves and colleagues [50] proved that higher optimism and lower general anxiety reduce fear of COVID-19.

Overall, in the pandemic context, the high levels of perceived stress were associated with high levels of hopelessness, depression, anxiety [51,52] and post-traumatic stress disorder [53], and all these symptoms along with fear and sleep problems were seen more frequently during the COVID-19 pandemic [54]. Previous research has shown the prevalence of anxiety in a lot of countries—in Germany: 50% [55], in Turkey: 45.1% [56], in China: 31.6% [57] and in Italy: 23.2% [58]. Recent findings argue that specific population groups are particularly at risk for developing negative mental health outcomes due to the differential impacts of the pandemic on these groups. Wang and colleagues [59] provide evidence that physical pain acts as a significant predictor of both depression and anxiety symptoms, and people with multiple disabilities (i.e., vulnerabilities) report higher anxiety symptoms. Women, people with pre-existing health conditions, people who have lost income and those in the lower-income categories report higher anxiety [51,58]. The absence of private space is also positively associated with depression and anxiety [60]. In addition, socio-economic disadvantages are associated with higher psychological distress and lower life satisfaction [61]. According to Magano and colleagues [62], fear and perception of this risk explain the impact of COVID-19 on travel in pandemic times, suggesting that the psychological impacts of fear and anxiety induced by the pandemic need to be handled as a public health priority. A higher level of COVID-19-related worry was significantly associated with a higher level of psychological distress. It is important to note that it is not only the trait anxiety (as an individual’s overall tendency) but also the specific state anxiety that color COVID-19 experiences of people from vulnerable groups. For instance, prevalence of health anxiety was found among women and individuals with chronic conditions [56]. There are certain psychological predictors of COVID-19-related anxiety such as emotional discomfort, depression, stress, COVID-19 fear, suicidal inclinations, resilience, neuroticism, optimistic expectations and social support [23,63,64,65,66]. However, to our knowledge, there is no systematic research on the combined effects of trait anxiety, resilience, perceived stress, optimism and pessimism on COVID-19-related anxiety in both the general population and vulnerable groups. Considering this gap, we conducted a study in the Bulgarian socio-economic environment to test these effects under the pandemic conditions.

*Research aim and scope:* Our study aims to explore psychological predictors of COVID-19-related anxiety in vulnerable groups during the pandemic in the Bulgarian socio-economic context. More specifically, we focus on the effects of anxiety and resilience (as personality traits), pessimism and optimism (as generalized expectations) and perceived stress as predictors of COVID-19 anxiety. In addition, we test the mediating role of state resilience in these effects. Research on specific groups with higher vulnerability can shed light both on the sources of fear and on the sources of resilience for them. The findings may serve to develop effective prevention-related and psychosocial support measures for these groups in society. A deeper understanding of vulnerable groups’ perceptions and responses to the COVID-19 crisis can help policy making and management strategies for future crisis situations.

**Figure 1:**
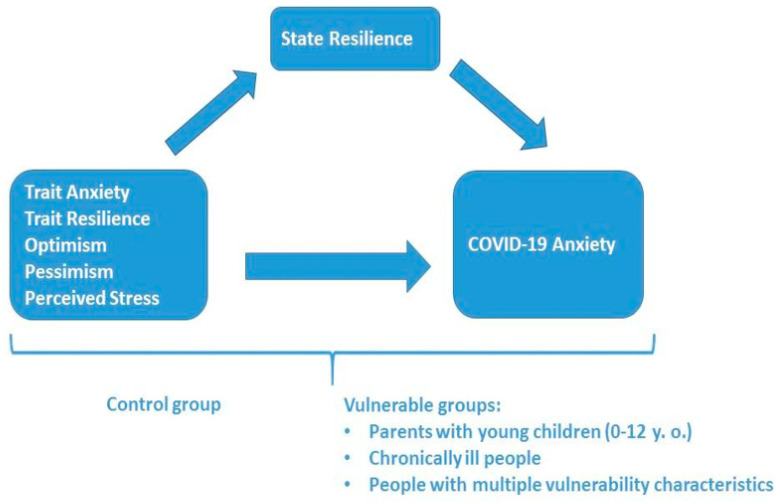
Research Model.

The research model suggests that there are specific psychological predictors of COVID-19-related anxiety and that state resilience, in particular, may mediate their effect on the anxiety caused by the global pandemic. The model was tested in concrete vulnerable groups (divided into three subgroups) and a control group with no self-reported vulnerabilities. The graphical representation of the tested research model is given in Figure 1.

The mediating effect of state resilience is tested on the grounds of the conceptualization of a simple mediation (process) model in statistics as one aimed at identifying and explaining a *mechanism* or *process* that underlies or intervenes a relationship between an independent variable and a dependent variable via the inclusion of a third hypothetical variable, known as a mediator variable (see Model 4, [67]).

### Research Hypotheses

**Hypothesis** **(H1).** *We assumed that trait anxiety, trait/state resilience, perceived stress and optimism/pessimism would be significant psychological predictors of COVID-19-related anxiety in the four studied groups—people with chronic physical conditions, working parents of young children, people with multiple vulnerabilities and the control group.*

**Hypothesis** **(H1.1).** *Between-group differences in the predictive power of the different factors over COVID-19-related anxiety were expected.*

**Hypothesis** **(H2).** *Findings from a qualitative study showed more positive emotions of parents with young children in comparison to chronic sufferers and those who lost their job/business due to the pandemic* [19]. *Therefore, we assumed that there would be differences in optimism/pessimism, anxiety, resilience and perceived stress between the four groups.*

**Hypothesis** **(H2.1).** *Based on previous research evidence* [57,58,61,68]*, we hypothesized that the group with multiple vulnerability characteristics would have the highest level of COVID-19-related anxiety, while the control group would have the lowest one.*

**Hypothesis** **(H3).** *We expected that state resilience would mediate (strengthen) the negative effect of optimism and trait resilience and (weaken) the positive effect of trait anxiety, perceived stress and pessimism on COVID-19 anxiety for every studied group.*

## 2. Materials and Methods

### 2.1. Participants and Procedure

To test the hypotheses, cross-sectional and intersectional quasi-experimental research was conducted in the autumn of 2022. An online survey via SurveyMonkey platform was conducted. Invitations for participation in the study were published and distributed on a number of media and social platforms such as the official websites of the Institute for Population and Human Studies—Bulgarian Academy of Sciences, the FB page and the website of the research project, the Bulgarian News Agency—BTA, etc. In addition, a targeted online campaign was made in order to distribute the survey invitations to special public and closed groups in the social media so as to reach representatives of the targeted vulnerable groups. Also, two of the biggest job centers (Rodopi and Blagoevgrad directorates) at the National Employment Agency e-mailed invitations to their clients (people registered as unemployed during the COVID-19 crisis).

The final data pull was composed of the following groups of people: (1) a control group (with no self-reported vulnerability) and (2) vulnerable groups, including (a) working parents with young children (0–12 y.o.), (b) people diagnosed with a chronic physical illness and (c) people with multiple vulnerability characteristics. Overall, 520 respondents aged 18–91 years old (*M* = 48.99; *SD* = 13.15), who were identified as belonging to only one of the four groups, participated in the study (all data available in Project “Socio-psychological Effects of the Crisis caused by COVID-19: Perceived Stress and Dynamics of Experiences” [69]). The frequency distribution and the sociodemographic profile of the respondents are represented in Table 1.

### 2.2. Measures and Scales

Five inventories were used:

- A short 3-item version of the Perceived Stress Scale (PSS) of Cohen, Kamarck and Mermelstein [70], a Bulgarian adaptation [14,71] that measured the degree to which people perceived their experiences “in the last 2 weeks” as stressful, unpredictable and uncontrollable on a 5-point Likert scale (1—It did not happen at all; 5—It happened very often, nearly every day). The following items were included: “In the last two weeks, how often have you felt that you were unable to control the important things in your life?”, “In the last two weeks, how often have you felt that things were going your way?” and “In the last two weeks, how often have you felt difficulties were piling up so high that you could not overcome them?”. Cronbach’s Alpha for the 3-item PSS was 0.70. The single-component structure was confirmed using PCA with 63.3% of the variance explained, as well as moderate inter-item correlations (0.61 > *r* > 0.34).
- Scales for Optimistic and Pessimistic Expectations—short versions of Scales for Generalized Expectations [72] were used based on previous publications [73]. Each scale consists of three items: “I’m a person who believes that there is a good way out of difficulties“, “I’m a person who looks at life optimistically“ and “I’m convinced that bad situations don’t last long“ (optimistic expectations, Cronbach’s α = 0.83) and “I’m more prepared for the worst because I think that it happens more often“, “There are very few pleasant things in life“ and “I think that one unpleasant thing usually leads to another” (pessimistic expectations, Cronbach’s α = 0.73). The statements were evaluated on a 5-point Likert scale (1—strongly disagree; 5—strongly agree). The two-factor structure was confirmed using PCA with 69.9% of the variance explained. Moderate-to-strong inter-item correlations (0.65 > *r* > 0.58 for the optimistic expectations scale; 0.57 > *r* > 0.38 for the pessimistic expectations scale) were found.
- A short 9-item version of the State–Trait Assessment of Resilience Scale (STARS) [74]. The original STARS is a 13-item scale with a 5-point Likert scale (1—disagree; 5—strongly agree) where state resilience refers to a person’s experience in a given situation, whilst trait resilience is a stable personality characteristic. Cronbach’s Alpha for the nine items was 0.89, for resilience state—α = 0.87 and for resilience trait—α = 0.77. The two-component structure was confirmed using PCA with 65.2% of the variance explained. Moderate-to-strong inter-item correlations (0.55 > *r* > 0.38 for the trait resilience scale; 0.78 > *r* > 0.47 for the state resilience scale) were found.
- A short version of the Spielberger state–trait anxiety inventory: Anxiety (Trait) [75]—the trait anxiety measures how one generally feels with 5 items on a 4-point Likert scale (1—not at all; 4—very much so). Cronbach’s Alpha for this study was 0.88. The single-factor structure was confirmed using PCA with 67.9% of the variance explained, as well as moderate-to-strong inter-item correlations (0.77 > *r* > 0.50).
- An 8-item version of the COVID-19 Anxiety Syndrome Scale (C-19ASS) by Ana V. Nikčević and Marcantonio M. Spada [76]—the original scale consists of nine items with 5-point Likert scale (0—not at all; 4—nearly every day). C-19ASS measures the way people cope with the COVID-19 threat. Cronbach’s Alpha for the eight-item version was 0.90. The single-component structure was confirmed using PCA with 58.2% of the variance explained, as well as moderate-to-strong inter-item correlations (0.68 > *r* > 0.39).

For the latter three, back and forth translation (English–Bulgarian and vice versa) was applied and scales were adapted for Bulgarian context by an expert committee.

Along with some additional questions aimed at collecting sociodemographic data, such as gender, age, education, occupation, income, etc. (see the data file on OSF [69]), the respondents were also asked 3 questions to identify their group belonging: “Have you been diagnosed with chronic physical illness (e.g., cardiovascular, pulmonary, oncological, renal, neurodegenerative, autoimmune, endocrine and/or metabolic)?” to identify people with chronic physical conditions; “Are there any 0–12 y.o. children in your household towards whom you exercise parental/guardian rights?” to recognize the parents with little children; and “In the last two years, have you lost a job/business due to the crisis caused by COVID-19? (e.g., if you were unable to perform a job/do business)?” to identify jobless/financially insecure participants. The group of people with multiple vulnerabilities composed of participants who responded positively to two or all three questions, while the control group composed of those who responded negatively to all three questions. As far as the groups of working parents with young children and chronic sufferers are concerned, only data for respondents, who were identified as belonging to either group, were analyzed. 

### 2.3. Statistical Methods and Procedure

The data were processed using SPSS v25, including the PROCESS v4.1 macro for mediation, moderation and conditional process analysis [67]. Firstly, we used reliability analysis (Alpha model), factor analysis (the method of principal components with Varimax rotation, including KMO and Bartlett’s test of sphericity) and inter-item correlation analysis (Pearson) to test the psychometric properties of the scales. Secondly, we proceeded with descriptive statistics, incl. data normality tests (skewness and kurtosis within +/−1), to test the data distribution. Thirdly, we employed Levene’s test for homogeneity of variances and parametric tests (ANOVA for normal data distribution and Scheffé post hoc test for homogenous variances) to examine the assumption about significant between-group differences in anxiety, resilience, perceived stress, optimism and pessimism. Fourthly, we applied multiple linear regression (stepwise method) with collinearity diagnostics (condition index < 30) to test our hypothesis about between-group differences in the effects of the studied traits and states on COVID-19 anxiety. Finally, we used Model 4 [67] of the PROCESS macro for SPSS and set state resilience as a mediating variable to examine if and how it intervened the effects of the fittest predictors on COVID-19 anxiety for every studied group.

## 3. Results

A descriptive analysis showed that the data distribution could be considered normal (see the descriptives in Table 2). Homogeneity of variances was observed (see Levene’s test results in Table 2). Quite surprisingly, ANOVA results showed significant between-group differences only for the pessimistic expectations—*F*(3, 518) = 4.06, *p* = 0.00—but not for the other factors (see all *F-* and *p*-values in Table 2). Using Scheffé’s pairwise test, we found that chronic sufferers reported significantly higher pessimism compared to the working parents of young children. Significant differences between them and the other two groups (the group with multiple vulnerability characteristics and the control group), as well as between the latter groups, were not observed (see pairwise tests in Table 2).

To scrutinize between-group differences in the predictive power of the different factors (trait anxiety, trait resilience, perceived stress, optimism and pessimism) on COVID-19-related anxiety, we applied Multiple Stepwise Regression with collinearity diagnostics. The results indicated that the inclusion of optimism and pessimism in the regression models, along with the other independent variables, did not have a significant effect except for optimism in the control group. The fittest models for explanation of COVID-19 anxiety in the four groups were as follows (see Table 3):

- *For chronic sufferers*, only trait anxiety was found to be a weak positive predictor—*R*^2^ = 0.079, *F*(1, 145) = 12.36, *p* = 0.001—which explained 7.9% of the variance in COVID-19-related anxiety.
- *For working parents with young children*, only perceived stress turned out a weak positive predictor—*R*^2^ = 0.049, *F*(1, 133) = 6.78, *p* = 0.010—which explained 4.9% of the variance in COVID-19-related anxiety.
- *For the group of respondents with multiple vulnerability characteristics*, both trait anxiety and perceived stress were found to be moderate positive predictors—*R*^2^ = 0.234, *F*(2, 138) = 20.74, *p* = 0.000— which together explained 23.4% of the construct variance.
- *For the control group*, trait resilience turned out to be a moderate negative predictor—*R*^2^ = 0.269, *F*(1, 99) = 36.12, *p* = 0.000—which explained 26.9% of the variance. At the same time, optimism and perceived stress together explained 32.8% of the variance in COVID-19 anxiety—*R*^2^ = 0.328, *F*(2, 99) = 23.68, *p* = 0.000. It is important to note that the main effect of trait resilience was studied separately from the other factors due to multicollinearity (condition index > 30).

**Figure 2:**
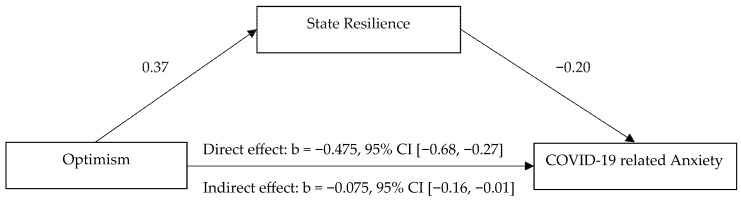
State resilience as a mediator of the effect of optimism on COVID-19-related anxiety in the control group (*n* = 100).

Finally, we carried out mediation analyses using PROCESS macro to examine if state resilience would mediate the effect of the fittest predictors of COVID-19 anxiety, i.e., trait resilience, optimism and perceived stress for the control group; trait anxiety and perceived stress for the three vulnerable groups (see Figure 2).

Our findings for the control group showed that state resilience significantly mediated only the effect of optimism, but neither trait resilience nor perceived stress, on COVID-19 anxiety. It strengthened the negative effect of optimism on COVID-19 anxiety and hence, helped to relieve the state anxiety of those who did not belong to any of the studied vulnerable groups. However, state resilience was not found to mediate the effect of trait anxiety nor perceived stress on COVID-19 anxiety in the vulnerable groups.

## 4. Discussion

Our main assumption (H1) that trait anxiety, trait/state resilience, perceived stress and optimism/pessimism will be significant psychological antecedents of COVID-19 anxiety in the four studied groups is partially supported as far as trait anxiety and perceived stress are concerned. Trait resilience and optimism were found to have significant effects on COVID-19-related anxiety only for the control group, while pessimism proved to have significant effect only for the group with multiple vulnerability characteristics. These findings come to support our hypothesis (H1.1) that the effect of these psychological factors will differ in the four groups included in our study. Positively, pessimism could not directly induce COVID-19 anxiety in the control group. Negatively, trait resilience and optimism of the studied vulnerable groups did not have a direct relieving effect on their COVID-19 anxiety and hence, some other mechanisms/processes/conditions, which mediate and strengthen this relationship, should be examined and introduced into clinical practice in the future to alleviate pandemic-related anxiety.

Significant between-group differences were observed only for the pessimistic expectations, with chronic sufferers having higher pessimism compared to the working parents of young children. This result supports previous qualitative research evidence [19]. As we did not observe significant differences between the other groups, our hypothesis (H2) was partially supported. Optimism also showed some tendency towards between-group differences, yet they were insignificant. Possible explanations for obtaining pessimism, but not optimism, as a clearly distinctive factor only between the chronic sufferers and the parents could be, on one hand, the highest mean age of the chronic sufferers as opposed to the lowest mean age of the parents of young children (see Table 1), combined with ill health, pain and/or poor health prospects of the former; on the other hand, pessimism and optimism are related constructs, but not a single two-dimensional construct, i.e., differences in/absence of optimistic expectations should not necessarily imply differences in/presence of pessimistic expectations and vice versa.

Our expectation that in contrast to the control group, the group with multiple vulnerabilities would have the highest level of COVID-19 anxiety (H2.1) was not met by the results of our study. Similar state anxiety relating to COVID-19 was found for all studied groups, including the control one. This finding suggests that healthcare measures would better address the psychological predictors and mechanisms of COVID-19 anxiety in the different groups rather than try to handle only the emergent state anxiety in times of crisis. 

Obviously, some psychological predictors of COVID-19 anxiety—trait anxiety and perceived stress—played a more significant role in the group with multiple vulnerabilities, compared to the other vulnerable groups—chronic sufferers and working parents with young children—where the same factors were observed, but as single weak predictors. These findings suggest that multiple vulnerabilities is related to more complex and stronger mechanisms of pandemic stress induction, which should be timely and simultaneously addressed in both healthcare and social policy making. At the same time, in the control group, trait resilience and the combination of optimism and perceived stress proved to be powerful predictors of COVID-19-related anxiety. These results highlight the importance of developing group-specific measures that should address mostly the trait anxiety of people with multiple vulnerabilities and chronic physical conditions, as well as the perception of stress for all groups, especially working parents with young children. Therefore, the measures for handling the sources of pandemic-related anxiety in people with multiple vulnerabilities and chronic physical illnesses should be year-round and long-term, while those for working parents of children 0–12 y.o. may not be so long-term, but should certainly consider their individual perceptions of stress under crisis conditions. Furthermore, the findings suggest that interventions for the vulnerable groups should focus mostly on handling their negative tendencies and perceptions, while those for the non-vulnerable groups should focus mostly on enhancing their positive psychological tendencies (i.e., trait resilience and optimism). These might be achieved through group-specific targeting and avoiding controversial messages sent by the responsible authorities, the media and various experts on the topics related to the threats and risks under the pandemic situation. Furthermore, the media reflections of the crisis caused by COVID-19 should have conveyed positive messages and problem-solving opportunities rather than negative messages through “apocalyptic” representations of the pandemic facts and future prospects.

Our last hypothesis (H3) that state resilience would mediate (strengthen) the negative effect of optimism and trait resilience and (weaken) the positive effect of trait anxiety, perceived stress and pessimism on COVID-19 anxiety for every studied group was partially supported. State resilience was not found to mediate the effect of trait anxiety nor perceived stress in the three vulnerable groups included in the study. These results suggest that clinical interventions, aimed at handling the negative effect of perceived stress and trait anxiety as a personality tendency on pandemic anxiety through enhancement of trait resilience, would not be effective. However, we found evidence that state resilience mediated the negative effect of optimism on COVID-19 anxiety in the control group, i.e., it helped to counteract the pandemic-related anxiety of those with no self-reported vulnerability. This finding prompts that fostering state resilience of people with no self-reported vulnerability may reduce the pandemic-related anxiety in healthcare and social care settings. In the context of previous studies on the relationship between anxiety and optimism [46,48,49,50], our findings outline some additional (non-)vulnerability-related aspects. Also, in line with previous research on resilience [37,38], our findings support the important role of resilience in relieving anxiety. Nonetheless, this highlights the need to regard resilience as a two-dimensional construct, including both trait and state resilience.

Overall, the results of this study emphasize the crucial importance of perceptions, expectations and anxiety (as a personality trait) to handle negative states and emotions in crisis times. They stay in line with previous findings [30] and also suggest that the concept of vulnerability must be regarded from multiple perspectives and that intersectional studies can contribute to gain a deeper understanding both for specific sources of stress and for certain directions on how different groups affected by crises may cope with it.

In line with Kuran and colleagues’ suggestion [77], we consider the results of a context-specific analysis—as those presented here—useful for both crisis and risk managers. On one hand, we find possible implications for building policies and measures designed with concern for the special needs and accessible resources of the vulnerable groups as well as their cultural characteristics. A better understanding of the specific stressors can help media and public communicators to adjust their messages to the perspective of specific groups in times of crisis. On the other hand, a more detailed picture of experiences of vulnerable groups can help the larger public to gain a deeper understanding of their specific perceptions and responses. Last but not least, frontline workers, responsible authorities, employers and representatives of the public sector as well as specialists in the field of psychosocial support could benefit from the findings in cases of future critical situations, when they need to quickly identify vulnerable groups in society and adequately address their needs.

*Limitations and implications for future research:* The presented results have certain limitations due to the self-reported online method of data collection. Since the sample is not balanced in terms of gender, age, education and occupational status, the influence of these and perhaps other factors remains beyond the scope of our analysis. Also, it is important to emphasize that the indicators of vulnerability, controlled in this study, are far from exhaustive for this concept and do not cover all its variations. The study was conducted in the autumn of 2022 in the Bulgarian socio-economic context when the crisis, caused by COVID-19, was in its advanced phase. The effects we observed might differ in their size and manifestation when it comes to the first waves after the outbreak or to the post-pandemic period. 

Nevertheless, our findings prompt some future research implications such as more comprehensive studies on multiple vulnerabilities (controlled for more factors and mechanisms), as well as inclusion of other groups and types of vulnerabilities (e.g., people diagnosed with mental health disorders; frontline workers; groups of people whose access to healthcare services is somewhat hindered such as marginal groups, refugees and asylum seekers; representatives of migrant, ethnic, religious or social minorities, etc.). Identification of additional psychological predictors and mechanisms of COVID-19-related anxiety would be very important for healthcare policy making and clinical practice. From the cross-cultural perspective of social psychology and related fields, future research may look for a replication of the studied effects in other socio-economic and cultural contexts.

## 5. Conclusions

In this study, COVID-19-related anxiety of people with no indicated vulnerability proved to be similar to that of vulnerable groups. With regard to psychological predictors and mechanisms of COVID-19 anxiety, several key points could be outlined:

- State resilience mediated the negative effect of optimism on COVID-19 anxiety and helped to alleviate it in the group with no self-reported vulnerabilities.
- Trait anxiety and perceived stress had a more significant role in the group with multiple vulnerabilities, compared to the other vulnerable groups—chronic sufferers and working parents with young children.
- Significant between-group differences were observed for pessimism. Chronic sufferers had higher pessimism compared to the group of working parents with young children.
- Trait resilience and optimistic expectations for the studied vulnerable groups did not have a direct relieving effect on their COVID-19 anxiety, while pessimism did not directly exacerbate the COVID-19 anxiety in the group of people with no indicated vulnerability.

The findings suggest that in crisis times, there should be group-specific measures aimed mostly at handling negative psychological tendencies and perceptions of vulnerable groups and strengthening positive tendencies and expectations of people with no indicated vulnerability. Public policies and measures, as well as healthcare interventions, should address the psychological predictors and mechanisms of COVID-19 anxiety in the different groups rather than attempt to tackle only the emergent (state) anxiety in crisis times.

## Figures and Tables

**Table 1:** Sociodemographic profile of the participants in the study.

Variable		Parents with Young Children	Chronically III People	People with Multiple Vulnerability Characteristics	Control Group	Total
*N* of respondents		134	146	140	100	520
*%*		25.8	28.1	26.9	19.2	100.0
Age	*M*	37.68	59.56	43.72	52.00	48.99
	*SD*	6.99	10.27	11.28	12.42	13.15
Gender	Male	14	14	14	16	58
	Female	131	118	126	81	456
	Missing/NA	1	1	0	3	5
Education	Primary	0	3	2	0	5
	Secondary	19	46	35	25	125
	Higher	115	97	103	75	390
Occupation	Manager	40	17	21	18	96
	Non-manager	62	38	52	33	185
	Student	1	0	3	1	5
	Retired	1	63	11	20	95
	Unemployed	10	9	23	11	53
	Other	18	17	26	13	74

Source: Authors’ analyses in SPSS (2022–2023).

**Table 2:** Descriptives and between-group differences for trait anxiety, COVID-19 anxiety, trait and state resilience, perceived stress, optimism and pessimism.

Factor	Group	M (SD) 95% CI [LL; UL]	Sk./K.	F (df 3) (Sig.)	Homogeneity of Variances-Levene (Sig.)	Pairwise Tests-Scheffé
Trait Anxiety	Chronic sufferers	2.529 (0.743) [2.408; 2.651]	−0.200/−0.613	0.379 (0.768)	0.384	n/a
	Parents of 0–12 y.o.	2.480 (0.749) [2.352; 2.608]	0.279/−0.441			
	People with multiple vulnerability	2.523 (0.811) [2.388; 2.659]	0.119/−0.869			
	Control group	2.434 (0.786) [2.279; 2.591]	−0.040/−0.647			
COVID-19 Anxiety	Chronic sufferers	2.043 (0.839) [1.905; 2.180]	0.770/−0.209	1.477 (0.220)	0.086	n/a
	Parents of 0–12 y.o.	1.969 (0.839) [1.826; 2.113]	0.823/0.168			
	People with multiple vulnerability	2.122 (0.995) [1.956; 2.289]	1.001/0.820			
	Control group	1.896 (0.812) [1.735; 2.057]	0.301/0.109			
Trait Resilience	Chronic sufferers	3.192 (0.575) [3.098; 3.286]	−0.857/0.538	0.280 (0.840)	0.084	n/a
	Parents of 0–12 y.o.	3.166 (0.483) [3.084; 3.249]	−0.193/0.238			
	People with multiple vulnerability	3.176 (0.586) [3.078; 3.274]	−0.240/−0.682			
	Control group	3.127 (0.553) [3.018; 3.237]	−0.327/0.360			
State Resilience	Chronic sufferers	2.955 (0.691) [2.843; 3.068]	−0.382/−0.096	1.100 (0.349)	0.138	n/a
	Parents of 0–12 y.o.	3.023 (0.604) [2.920; 3.126]	−0.645/0.783			
	People with multiple vulnerability	2.878 (0.673) [2.766; 2.991]	−0.055/−0.620			
	Control group	2.966 (0.692) [2.829; 3.103]	−0.255/−0.428			
Perceived Stress	Chronic sufferers	2.412 (0.892) [2.266; 2.558]	0.623/0.206	1.571 (0.196)	0.302	n/a
	Parents of 0–12 y.o.	2.403 (0.933) [2.243; 2.563]	0.621/−0.087			
	People with multiple vulnerability	2.620 (0.977) [2.457; 2.784]	0.409/−0.385			
	Control group	2.4883 (0.998) [2.290; 2.687]	0.415/−0.432			
Pessimism	Chronic sufferers	2.897 (0.910) [2.748; 3.046]	−0.171/−0.408	4.055 (0.007)	0.111	ChS > P **
	Parents of 0–12 y.o.	2.531 (0.865) [2.383; 2.679]	0.159/−0.092			
	People with multiple vulnerability	2.801 (1.011) [2.631; 2.971]	0.307/−0.498			
	Control group	2.670 (0.905) [2.491; 2.850]	0.125/−0.580			
Optimism	Chronic sufferers	4.043 (0.805) [3.912; 4.175]	−0.803/0.606	2.569 (0.054)	0.255	n/a
	Parents of 0–12 y.o.	4.109 (0.654) [3.998; 4.221]	−0.197/−0.833			
	People with multiple vulnerability	3.896 (0.871) [3.751; 4.043]	−0.682/0.382			
	Control group	4.161 (0.750) [4.013; 4.311]	−0.765/0.103			

Note: M = Mean value; SD = Standard deviation; CI = Confidence interval at 95%; Sk. = skewness; K. = kurtosis; Significance ** *p* < 0.01; ChS = chronic sufferers; P = Parents with little children 0–12 y.o.

**Table 3:** Fittest predictive psychological models of COVID-19-related anxiety in the four groups: the effect of trait anxiety, perceived stress, trait resilience and optimism.

				95% CI			
Model		Beta	*SE*	*LL*	*UL*	β	*p*
**Chronic sufferers**							
1.	Trait Anxiety	0.317	0.090	0.139	0.496	0.281 *	0.001
**Parents with little children**							
1.	Perceived Stress	0.199	0.076	0.048	0.349	0.221 *	0.010
**People with multiple vulnerabilities**							
1.	Trait Anxiety	0.536	0.092	0.354	0.718	0.445 *	0.000
2.	Trait Anxiety	0.377	0.110	0.158	0.595	0.313 *	0.001
	Perceived stress	0.228	0.091	0.049	0.408	0.230 *	0.013
**Control group**							
1.	Trait Resilience	−0.762	0.127	−1.013	−0.510	−0.519 *	0.000
2.	Optimism	−0.443	0.096	−0.634	−0.252	−0.409 *	0.000
	Perceived Stress	0.229	0.072	0.086	0.373	0.282 *	0.002

Note: Multiple linear regression (stepwise method); Beta = unstandardized coefficient; SE = Standard error; 95% CI = confidence interval at 95%; *LL* = lower limit; *UL* = upper limit; β = standardized coefficient; * *p <* 0.05.

## Data Availability

Research data and materials can be found at https://osf.io/hrw78/ with https://doi.org/10.17605/OSF.IO/HRW78.
